# Honey Bee Foraging Decisions Are Shaped by Floral Trait Distinctiveness and Perception of Gains or Losses

**DOI:** 10.3390/insects16090884

**Published:** 2025-08-25

**Authors:** Juan C. Hernández, Jair E. García, Harrington Wells, Marisol Amaya-Márquez

**Affiliations:** 1 Instituto de Ciencias Naturales, Universidad Nacional de Colombia, Bogotá 111321, Colombia; juchernandezpe@unal.edu.co; 2 School of Physiology, Monash University, Melbourne, VIC 3800, Australia; jirgarci@gmail.com; 3 Health & Natural Sciences, Biological Science, University of Tulsa, Tulsa, OK 74104, USA; harrington-wells@utulsa.edu

**Keywords:** bee cognition, honey bee foraging ecology, bee decision making, flower distinctiveness, opportunity cost, loss aversion

## Abstract

The floral choices of honey bees (*Apis mellifera*) were studied using artificial flower patches to understand how foragers manage changing floral landscapes. Bees were observed under conditions where reward quality changed over time in blue and white flowers. We evaluated initial learning and reversal learning, varying the magnitude of reward quality-difference and color distinctness in the honey bee’s color vision space (being either similar or more distinct). Flower color fidelity was higher when flower colors were more distinct, but it also made it more difficult for bees to abandon the flower color in the reversal learning phase. Smaller differences in reward quality reduced flower color fidelity and promoted reversal learning. When reward difference between flower colors was created (initial learning), a decrease in one of the flower color rewards elicited a stronger behavioral response from foragers than an increase in reward. Our work highlights that bees used and integrated information from different axes of information: distinctiveness of color cues, magnitude of reward difference, and directionality (being stronger for losses than gains). Thus, flower distinctiveness, opportunity cost, and loss aversion drive honey bee foraging decisions. Higher accuracy at initial learning has stronger costs in behavioral adaptations at changing floral landscapes.

## Simple Summary

Bee foraging decisions are complex cognitive processes, given that foragers need to constantly compare both cues and rewards between alternative floral choices. Floral specialization (fidelity) to the best option is the optimal behavior, but loses value as floral market offers change. Here, in this study, we investigated how forager honey bees deal with floral choices in a changing floral landscape. Does the degree of flower distinctiveness affect bees’ accuracy in floral choice? Do bees perceive losses differently to gains? The perception of phenotypic distance between flowers, as well as the direction of nectar production (increase/decrease) affected honey bees’ accuracy in selecting the correct flower color. Foragers developed flower fidelity to the color offering the higher nectar concentrations in each experiment. However, learning accuracy was not the same for all the differences in reward value, indicating that foragers scale between options, using both color cues and differences in reward quality. The more difficult discriminations have a longer reversal ratios, a signature of cognitive costs in adaptive foraging to changing floral landscapes. The mechanism underlying decision-making in honey bee foragers includes opportunity cost and loss aversion. The costs of cognitive specialization were evident in behavioral adaptation.

## 1. Introduction

The relationship between insect pollinators and flowers has been framed as a specialized mutualism [1,2,3,4,5,6,7,8,9] where the evolutionary specialization of pollinators of plant species has resulted from natural selection. While plant and pollinator species have their own species-level specialization, their interactions do not occur in a vacuum and the floral landscape continuously changes, even within the foraging season of an insect pollinator. These changes lead to decision scenarios involving flower availability and pollinator cognitive abilities. The ecological scenario in which this interaction occurs has been compared with a floral market where plants offer rewards and bees choose which flowers to specialize on at a particular time [10,11,12,13,14]. In this context, decisions are manifested as behaviors which allow foragers to develop ecological specialization in the use of particular spatial-temporal floral resources [15].

Foraging decisions are based on learning to associate floral traits with rewards by successive differential conditioning [16,17] and subsequently the cognitive capacity to solve the environmental-resource problem that is manifested (e.g., [18,19,20,21,22,23,24]). In fact, foraging decisions have long-term consequences on an individual’s energy budget, which led to the energy-maximization model, where average net energy gain (kcal/hour) is expected to drive foraging decisions [25]. Nevertheless, eusocial species act as a “super organism” and the colony at times takes precedence over what would be best for individuals [26,27]. This is manifested in honey bees when encountering a high-quality nectar source, which foragers do indeed choose over lower-quality rewards; however, foragers take back a partial crop load of nectar at least initially [28]. The colony ultimately gains through the speed of recruitment of naïve bees to the extra ordinary energy resource. Here, the consequence for the colony is magnified by competition with other pollinators via a race to capture these fleeting assets. The eusocial aspect of foraging also manifests itself in the crop-attached forager-constancy observed in the face of superior rewards (e.g., by Wells et al. [19,29]), presumably on the colony level to control all nectar energy sources, leaving little for competitors.

Furthermore, the result of poor decisions is magnified by competition with other pollinators for the same floral resources via a race to capture these fleeting assets. Moreover, learning can be viewed as expensive [30], even when solving simple associative tasks.

Time-related cost increases when distinguishing between flowers is difficult (e.g., when flower colors are similar). This cost is expressed in the time it takes to make a choice and in the number of mistakes made [31]. Further, complex problems lead to different foragers solving the same problem in different manners. In floral problems involving great discrepancies in floral reward and the work required to glean the nectar reward, three different solutions were seen in populations of honey bee foragers [32]. Similarly, Dyer and collaborators [33] found that in reversal learning, three types of “bee personalities” emerge when flower color distinction was difficult. In both studies, one type quickly responded to changes in rewards associated with cues, and another group was unable to. In terms of work and energy, this translated into a set of bees that maximized energetics, choosing high reward (net energy maximization, the ‘deliberate decisive’ bees of [32,33]). The other extreme [32] were bees that remained loyal to flower color regardless of reward or cost (the ‘stay’ bees of Dyer et al. [33]). The third group minimized cost at the expense of a higher net reward. The results of our reversal experiment are also consistent with the performance of ‘deliberative-decisive bees’, forager bees that could make a change to learnt preferences [33]. In these experiments, bees that were able to reverse learning attained accuracy levels between 70% and 80% in fifteen trials following a reversal in the color of the rewarding target.

A dynamic floral environment imposes an extra cost to foragers by making acquired information outdated [30]. Nevertheless, the perception of current differences can be affected by previous experience [34]. In some cases, memories contribute to new decision scenarios through the use of information acquired from solving past foraging problems [35]. The use of search images based on previous associations between flower color and reward has even led bees to prefer the floral type with the lower reward in some instances [36]. Nevertheless, whether an apparently suboptimal foraging behavior is truly suboptimal depends on the factors affecting a behavioral decision [25]. The ways foragers assess, acquire, and use information when making a floral choice poses key questions in the field of cognitive ecology, such as how organisms manage multidimensional factors (e.g., [20,37,38,39,40,41]). A previous study showed that when differences between nectar rewards in alternative floral options are small, honey bees specialize in visiting the more distinctive flower color even though it is an energetically less profitable decision per flower [42]. Hence, the flower’s phenotype-reward association has the potential to affect plant–pollinator mutualism, both at the ecological scale by determining a degree of specialization in resource use, and at the evolutionary scale by shaping the cognitive architectures of forager bees [4,43]. As such, these differences can shape floral traits and phenological patterns [41,42,44,45,46,47], though patterns may differ between the ecosystems around the world [47]. Studies focused on either evolutionary, ecological, or both aspects of bees’ food specialization (e.g., [48]) will contribute information to expanding theories on the mechanisms underlying plant–pollinator mutualism.

How foragers assess reward quality is central to the understanding of floral choice (e.g., [20,49,50,51,52]). Once learning is consolidated in a honey bee worker’s memory, it will affect her behavior when the color cue does not match the expected nectar reward, even in novel situations. Thus, in addition to perceptions of floral cues and nectar rewards, memory of past associations is also important in making future decisions [34,35,50,51,52]. In this respect, reversal learning measures behavioral flexibility and is relevant to tracking foraging resources in a dynamic environment over time.

Reversal learning involves situations where stimulus–outcome or response–outcome relationships are switched, and it has been an important tool for comparing learning across taxa (e.g., [53,54,55]). Of relevance is the emerging picture that reversal learning is disrupted in many psychiatric and neurological disorders (e.g., [56,57,58]).

Reversal learning has many of the same attributes as classical conditioning, with the Rescorla–Wagner model [59] predictive of learning over time [53]. Classically, models depicted inhibitory control as the basis of reversal learning [59]. However, recent work with vertebrates suggests excitatory learning is involved [60] as well. Notably, reversal learning does not appear to be a simpler cognitive process in insects such as bees [61]. Ben-Shahar [62] demonstrated that in honey bees two dissociable processes constitute reversal learning: those inhibitory and those excitatory. Further, at least in bees, excitatory and inhibitory learning appears to involve different molecular processes [63].

The molecular process seems to be influenced by a host of factors, involving seasonality as well as cast. In fact, how protein synthesis inhibitors affect the process seems to be reversed in bees between summer and winter [63]. Further, significant differences in reversal learning occur even within bee taxa. Honey bees respond to the stimulus as it changes contingencies and factor in an average of past experiences with the same stimuli so that across successive reversals error rate increases [64]. In contrast, bumblebees reduced errors and improved performance across successive reversals [65].

In this study we investigated the cognitive flexibility of foraging honey bees in the context of learning and reversal learning under conditions involving different flower phenotypes where both primary floral traits (reward qualities) and secondary floral traits (color cues) were varied. We examined how current nectar reward difference, color distance, and direction of nectar production (increase/decrease) influence accuracy in floral choice, and in turn how it affects the perception of reward differences in future choices as it would occur in a dynamic floral landscape.

## 2. Materials and Methods

### 2.1. Materials

**Figure 1:**
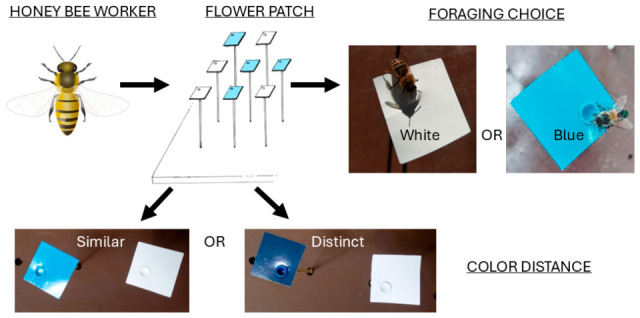
Schematic representation of the experimental workflow whereby honey bee workers were allowed to forage in artificial flower patches that consisted of blue and white flowers. The treatment groups varied in the difference in sugar concentration between colors as well as the distinctiveness of the colors themselves, being either more similar (near in color distance) or more distinct (closer in color distance), and the method to create differences in reward (increasing or decreasing with respect to the baseline value). Honey bee icon credit: DBCLS 統合TV, CC BY 4.0 <https://creativecommons.org/licenses/by/4.0>, accessed on 19 July 2025 via Wikimedia Commons.

The experiments examined the foraging decisions of European honey bees, *Apis mellifera* (Hymenoptera: Apidae), visiting artificial flowers in patches containing flowers of two different colors following the methodology of Wells et al. [19,24,66,67]. Each patch consisted of 36 artificial flowers, 18 white and 18 blue (to humans). The flowers were displayed randomly with respect to color in a 6 × 6 Cartesian array, with rows and columns 70 mm apart on a brown pegboard. Each artificial flower consisted of a 9 cm^2^ square plasticized paper (corolla) (Figure 1) containing a cylindrical tube 8 mm in depth (nectary), mounted on a 130 mm length pedicel. The climatic conditions at which the experiments were conducted were an average temperature of 18.12 ± 1.77 °C and an average relative humidity of 57 ± 10.4%. The diet model [25] was used to assess the economic decision-making of forager honey bees. We quantified each bee’s learning accuracy in flower choice in response to the differences in nectar concentration and color distance of floral options, and whether the bees perceived the direction of nectar production (increase or decrease). Thus, learned floral fidelity was the behavior that allowed us to see what information the bees were using to make economic choices.

### 2.2. Methods

Our study had two foci: the first was on how reward differences and flower color distinctness interacted to affect initial appetitive learning, and the second was on how they affected appetitive reversal learning.

Reward quality difference between flower colors was the main driver for forager decisions, and flower color the visual reward cue. We were interested in how three factors affected forager learning: (1) the magnitude of reward difference between flower colors, (2) the visual proximity of the two flower colors in the bee’s color vision space [68], and (3) whether the absolute difference in reward was created by increasing or decreasing the reward with respect to the baseline training value.

We conducted 16 experiments (Table 1). Each experiment consisted of three consecutive treatments: Treatment I offered foragers the same reward in both flower colors (baseline). Treatment II offered a higher-quality reward (sucrose concentration) than the alternative color (focus 1—learning). In Treatment III the flower color offering the higher-quality reward was switched (focus 2—reversal learning). Half of the time, blue flowers (B) offered the higher-quality reward in Treatment II, and the other half of the time, white flowers (W) offered the higher-quality reward. Each flower offered a 4 µL reward, and a flower was refilled immediately after a bee visited it. Under the climatic conditions in which the experiments were conducted, evapotranspiration of the nectar did not occur. Each treatment lasted until a bee made 5 round trips from the hive (5 × 3 treatments = 15 round trips per experiment). For each bee we counted a ‘flower-visit’ when a bee alighted on a flower and tasted the sucrose solution with either its proboscis or antennae. We recorded the sequence of flower colors visited by each bee to the flower patch. Each bee was used in only one experiment and was removed permanently from the experiment after completing 15 return trips. Only one bee was tested at a time. Non-focal individual bees were removed from the patch using a Well’s designed trap, which consisted of a long-ventilated wood framed and netted box (35 cm long × 15 cm wide). The trap was open at the base to facilitate the removal of bees from the foraging arena, and was set up to provide sucrose ad libitum via a removable Petri dish. At the end of the experiment, non-focal bees captured in the trap were released in good condition. Reward differences of (Δρ%) 10, 20, 30, and 40% (*w*/*v*) were presented to the test bees (four reward differences), but only one Δρ% per experiment, and a particular bee only was tested with one of the four reward differences (Table 1).

We created the reward difference in two different ways (two methods in which the rewards were changed). The first was by increasing the quality of the reward in one of the flower colors from that offered in Treatment I, while the alternative flower color’s reward remained the same as offered in Treatment I (gain). The second was by decreasing the quality of reward in one of the flower colors from that offered in Treatment I, while the alternative flower color’s reward remained the same as offered in Treatment I (loss).

**Figure 2:**
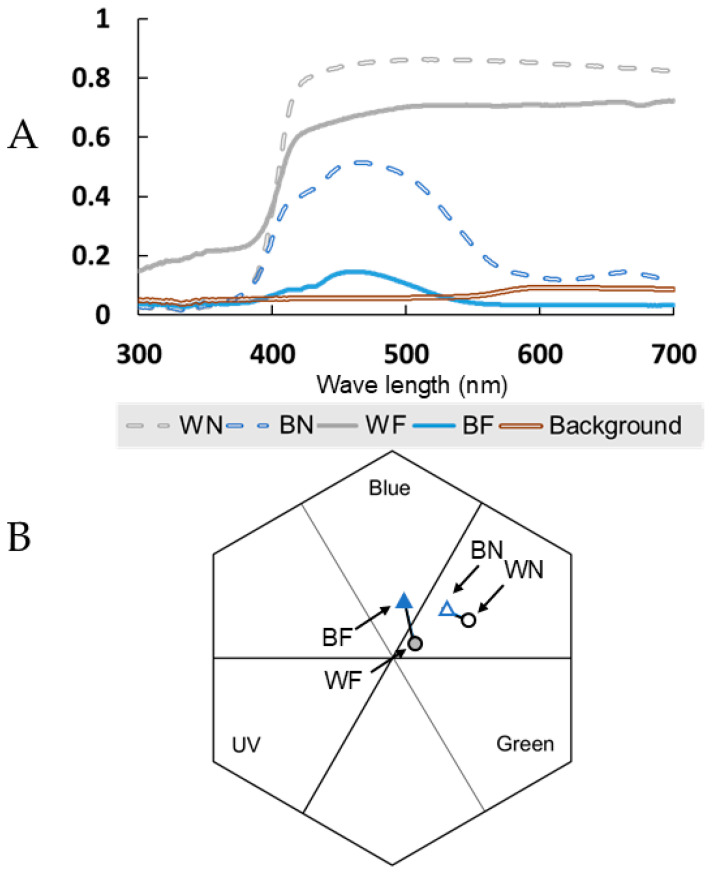
Flower colors used for the experiments. (**A**) Spectral reflectance of the two blue colors (blue lines) and two white colors (gray lines) plus the brown pegboard background. (**B**) The colors that are nearer in color distance are marked by dashed lines, the colors that are farther apart between them are marked by solid lines. WN = white near, BN = blue near, WF = white far, BF = blue far. B. Flower colors modeled in the honey bee color hexagon: “blue” triangles, “white” circles.

Finally, we explored the distinctness of the flower color choices (color–distance) in the bee color vision space [69] and learning. We used two different situations (two distinct binary colors). The first had a pair of colored flowers relatively “far” apart in the bee’s color space: 0.142 units in the Hexagon Model (Figure 2). The second situation had the pair of flower colors relatively “near” in the bee’s color space: 0.052 units in the Hexagon Model (Figure 2). Experiments 1 to 8 used the “far” (more distinct) flower color pair. Experiments 9 to 16 used the “near” (more similar) flower color pair. The colors in each pair could be discriminated by honey bees (see below: flower color modeling).

#### 2.2.1. Learning Data Analysis

Learning (focus 1) was analyzed using a repeated-measure MANOVA [69] on the results of Treatments I, II, and III. We recorded the color (blue vs. white) offering the greater reward in Treatment II, the reward difference (4 different Δρ%), color distinctness (far vs. near), and the method (increase vs. decrease reward), along with the treatment (3 repeated measures), and all cross-effects were tested.

#### 2.2.2. Reversal Learning Data Analysis

Treatment III was a test of reversal learning, as well as being an overall experimental control. Here, the rewards associated with the flower colors were reversed from that of Treatment II. Since the experimental conditions (reward difference, color distinctness, and method) resulted in differing levels of proficiency in the initial learning phase (Treatment II), we compared each bee’s performance in Treatment III to its initial learning in Treatment II. The degree of learning was measured as the difference from random flower visitation, i.e., (B%trt−50%).

The reversal ratio (rR) then was defined as

rR = − (B%trt3 − 50%)/(B%trt2 − 50%)(1)

and is a measure of how well a forager switched flower color preference in Treatment III when the rewards associated with the flower colors were reversed. Notice that reversal learning should result in a forager switching flower color preference from blue to white, or white to blue, between Treatments II and III, depending on the experimental trial. Thus, either the numerator or denominator will be negative, but not both.

A score of rR = 1 represents perfect reversal, while rR > 1 indicates better performance in the reversal task than in the initial learning. When 0 < rR < 1, reversal learning was poorer than initial learning. Negative rR scores, rR < 0, represent failure to switch flower colors between Treatments II and III.

There were 324 bees used in the set of experiments, of which 3 foragers (approx. 1%) failed in the initial learning test (Treatment II); they continued random flower selection in Treatment II and had a very high rR score due to learning in Treatment III. Since initial learning did not occur, reversal learning could not be measured in these bees. They were eliminated from the analysis of reversal learning.

Of the remaining 321 bees, there were two distinct populations in terms of rR scores. The first group was composed of 33 foragers (approx. 10%) and was characterized by each bee having rR < 0; reversal learning was never truly demonstrated by switching flower color fidelity. In the second group, each bee had rR > 0 (*n* = 288 bees), and this was the group used for statistical analysis.

Reversal learning (focus 2) was analyzed using an ANOVA [69] with the rR score and the following factors: color (Treatment III with greater reward), reward difference, color distinctness, and all cross-effects. The method of creating the reward difference in Treatment II does not apply, since rewards associated with color were simply reversed.

#### 2.2.3. Flower Color Modeling

The reflectance spectra from the artificial flowers and board background (Figure 2A) were recorded with an AGILENT Varian Cary 5000 UV-VIS-NIR spectrophotometer (Physics Department at the Universidad Nacional de Colombia, Bogotá, Colombia). Reflectance spectra were modeled in the hexagon color-model of [68] shown in Figure 2B, assuming a typical daylight illumination under an open sky equivalent to CIE D65 [70]. The spectral power distribution of the daylight spectra was expressed as quantum flux [71]. We used the reflectance spectra from the pegboard surface as the adaptation background for our calculations. The Euclidean distance between the points representing white and blue stimuli in the hexagon space were 0.052 and 0.142 (Supplementary File S1). Our modeling indicates that color differences between the various stimuli used in the experiment can be discriminated by honey bees with an accuracy higher than 99% under an appetitive–aversive differential conditioning [17,72], and with an accuracy of about 88% under absolute conditioning [72]. Therefore, the two stimuli used were easily discriminable by a foraging bee under our experimental conditions.

## 3. Results

### 3.1. Learning

**Figure 3:**
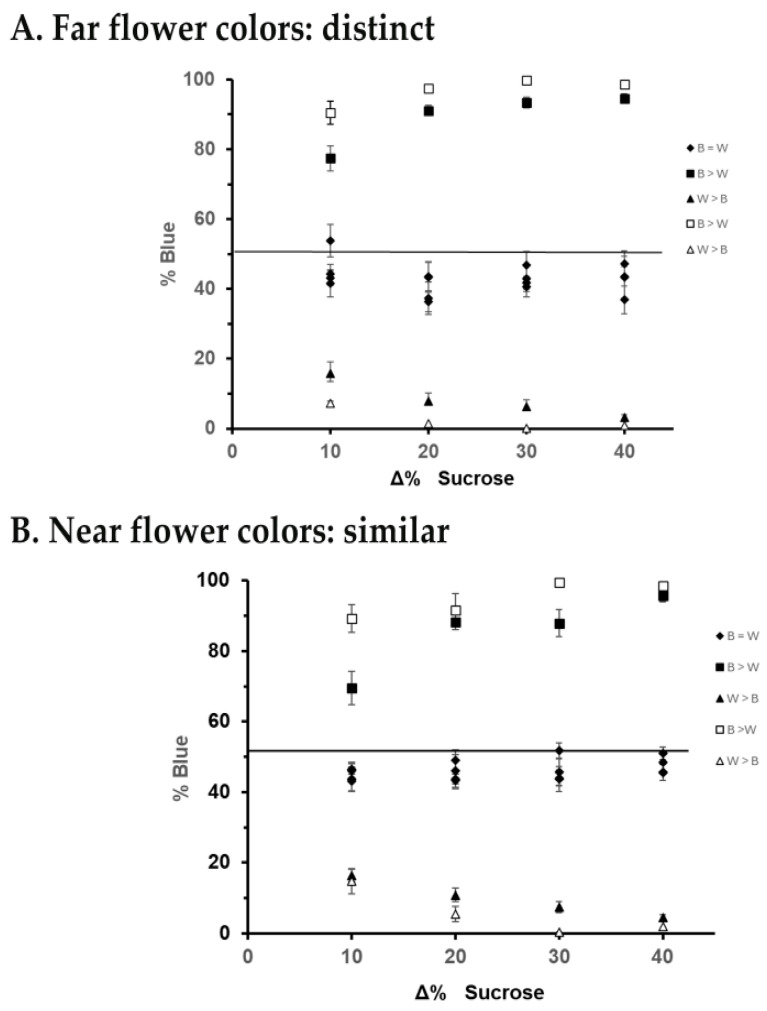
Results of focus 1: initial learning using the (**A**) far flower color dimorphism—distinct colors, and (**B**) near flower color dimorphism—similar. Presented are data from Treatment I (diamonds ♦) and Treatment II (squares and triangles) as the mean + se. Solid squares (■) and triangles (▲) represent the method where reward was increased in one flower color, and open squares (□) and triangles (Δ) where reward was decreased in one flower color. Squares (■, □) show results where blue flowers had the greater-quality reward, and triangles (▲, Δ) where white flowers had the greater-quality reward. Treatment I for each experiment is shown with the Treatment II reward difference.

The analysis of initial learning performance focused on floral color fidelity as a function of color distinctness, reward difference, and method of creating the reward difference in second training phase of the experiment (i.e., increase or decrease). Foragers visited flowers randomly with respect to color in Treatment I, where there was no difference in reward, and heavily favored the flower color offering the greater-quality reward in Treatment II (Figure 3), and then switched flower color fidelity when the rewards were reversed with respect to flower color in Treatment III. During each treatment, honey bees foraged throughout the floral patch (i.e., bees did not limit their nectar feeding to a particular area of the patch). The main factors driving forager flower choice were treatment (I, II, III) and color (with blue or white having the higher reward in Treatment II); the significant factors and interactions of the MANOVA analyses are displayed in Table 2.

Reward difference and color distinctness were significant factors with respect to forager flower color fidelity, albeit to a lesser extent (Table 2). As might be expected, a greater reward difference between flower colors resulted in foragers being more selective (Figure 3). When colors were more similar and thus more difficult to distinguish (less distance between them), bees showed less specialization in flower choice (Figure 3). Reward-difference x color distinctness x treatment was a significant interaction (Table 2). Also, reward difference x method x color was a significant interaction (Table 2). Bees were more accurate at choosing the color containing the greater reward when the alternative flower color had decreased in value than when the color containing the greater reward had an increase in reward concentration; this occurred at all values of reward difference (Figure 3). Other significant interactions are shown in Table 2. Forager bees visited between 15 and 20 flowers per round in the five rounds of Treatments I and II, while in Treatment III forager bees increased the number of visits per round to between 30 and 40 flowers during the two first rounds.

### 3.2. Reversal Learning

**Figure 4:**
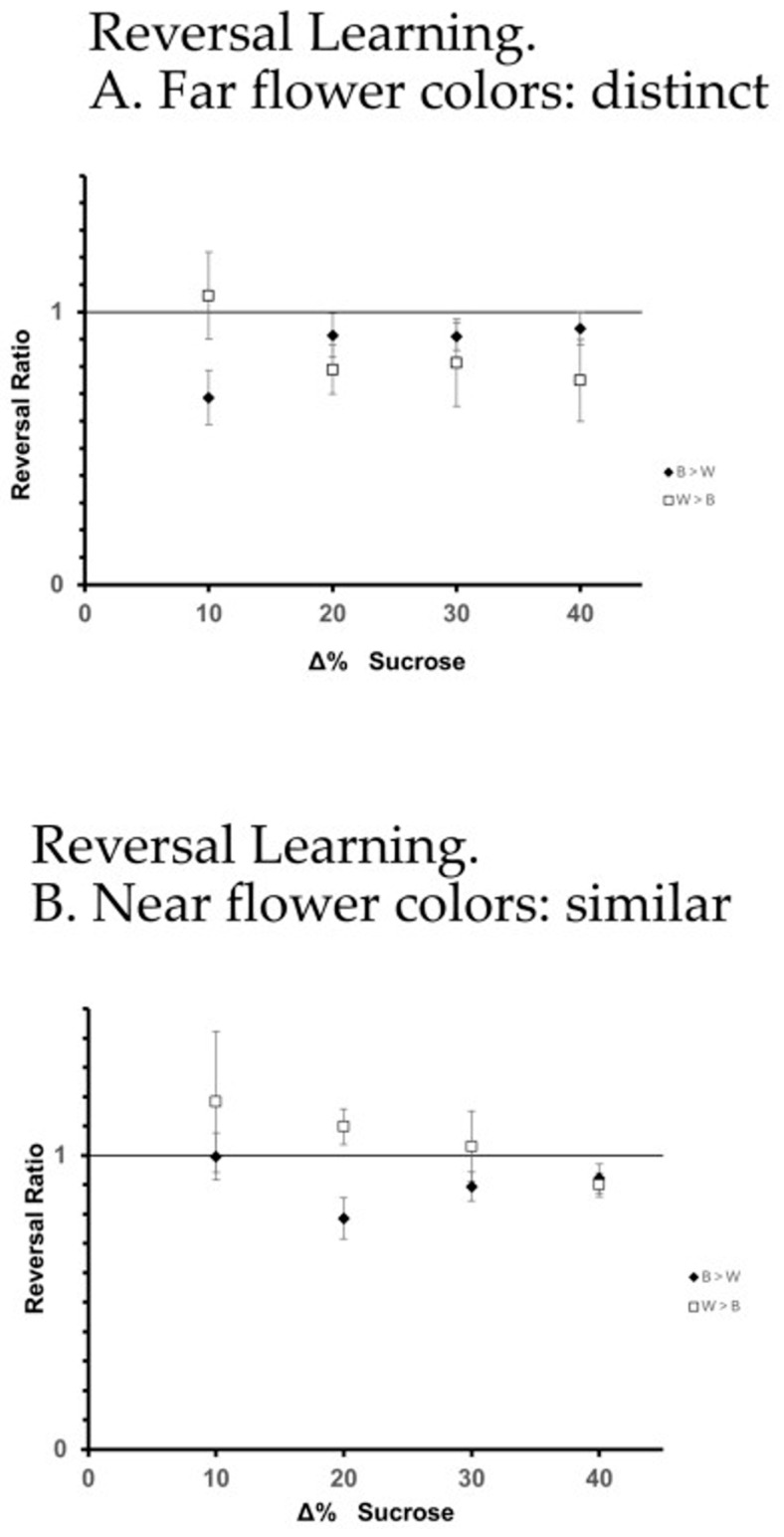
Results of focus 2: reversal learning using the (**A**) far flower color dimorphism—distinct colors, and (**B**) close flower color dimorphism—similar colors. Presented are the reversal ratio data (rR) as the mean + se. Diamonds (♦) indicate where Treatment III had blue flowers with the higher caloric reward, and squares (□) where white flowers had the higher caloric reward in Treatment III.

The analysis of reversal learning performance focused on quantifying how well a forager was able to reverse its flower preference relative to the previous training phase (2nd vs. 3rd training phase), and was conducted using the reversal ratio (rR). The factors that were significant in predicting the differences in rR (ANOVA) are flower color distinctness (far/distinct vs. near/similar: F1280 = 3.864, *p* = 0.050), and color x reward difference (F1280 = 4.248, *p* = 0.040). All other factors and interactions were not significant (*p* > 0.05). Surprisingly, bees did slightly better at reversal learning when flower colors were near (i.e., more similar; Figure 4), and they performed even better when the color containing the higher reward was white.

## 4. Discussion

Making optimal economic choices is a cognitive challenge for most animals, including humans, and is of time-sensitive importance for honey bee colonies that need to feed up to 60,000 sisters. In our study we used artificial flower patches that contained two types of flower colors, both of which offered a nectar reward, where we controlled whether white (W) or blue (B) flowers had a higher nectar concentration. Here we found that bees became more selective when the difference in reward offered by one of the two flower colors was greater. In all the experiments, honey bees changed foraging behavior, from random color choice (B = W in Treatment I, Figure 3) to specialized feeding in the flower color offering the greater reward (B > W or W > B in Treatment II, Figure 3), demonstrating that bees learned to choose the flower color with the greater reward among the two options. Although previous studies have reported learned flower fidelity based on economic choices both in social and in solitary bees (e.g., [29,37,67,73,74]), less is known about the mechanisms underlying this specialized behavior, which requires from the forager the capability to compare and rank the nutritional values of alternative options [25] to make the correct economic choice. However, the empirical results conform to the expectations of the Rescorla–Wagner model of conditioning [59] not only in bees favoring the flower color with the greater reward, but also in the strength of the association between flower color and reward. That is, Rescorla–Wagner’s model predicts that both more salient conditioning stimuli and rewards increase the association between flower color and greater reward. The result predicted is that bees will become more selective in flower visitation.

Direct comparison is an important step in the integration of information from a range of rewards for honey bees; however, honey bees are known to overestimate higher-quality rewards [24]. In the present study we used artificial floral patches resembling a “floral-market” with only two flower species for bees to choose from. In this scenario, just the perception of greater and lesser as distinguishable categories would suffice for a forager bee to specialize in the flower color offering the greater reward. If that would have been the case, we expected total preference for the greater option in Treatment II across all the 16 experiments. Instead, the results showed that the magnitude of the reward difference used in each experiment (Table 2), [(Δρ%) 10, 20, 30, and 40% (*w*/*v*)] mattered. Honey bees exhibited a pattern of floral choice marked by differential degree of flower fidelity, where higher deltas prompted higher accuracy (Figure 3), exactly as predicted by Rescorla–Wagner [59]. Greater difference in reward heightened the ultimate expectation of the unconditioned stimulus. Our finding results suggest that honey bees have the capacity to gauge the absolute magnitude of the difference in reward and use that quantitative information more so than qualitative information (categories: greater and lesser) to modulate the degree of cognitive attention paid in flower specialization. This result is also congruent with a previous report on the effect of the amount of reward on accuracy in honey bees [75].

Color distinctness between floral options was a significant factor in floral choice (Table 2). Flower color distance showed a consistent pattern, characterized by bees that overall did slightly better at color learning when foraging among more distinct flower colors than when they chose between more similar colors (Figure 3). This result also is consistent with the Rescorla–Wagner model [59], which predicts that the salience of the conditioning stimuli will lead ultimately to fewer choices of the lower reward. More distinct flower colors led bees to become more readily attached to the higher-rewarding flower type, while it was harder for bees to abandon a flower type during reversal learning. Previous studies have reported that costs increase when colors are more difficult to distinguish, both in ecological contexts, where deceptive flowers similar in color to those rewarding flowers acquire reproductive benefits from the forager’s difficulty to discriminate similar colors [13,17], and in laboratory contexts evidenced by difficulty in learning color discrimination, increasing the response time, or both, leading researchers to use a training paradigm of appetitive–aversive differential conditioning to train the bees (i.e., when there is a direct negative impact for making the wrong choice [17,23,53,59,76]. Our results showed that forager honey bees were able to discriminate between similar colors (“near”) under an appetitive–appetitive differential conditioning (i.e., when the suboptimal choice was still a benefit). This result is interesting in terms of economic choices, as it shows that although the bees were not receiving a direct physiological punishment in the alternative color to the rewarding one (e.g., less sugar as opposed to quinine, water, empty flowers, electrical shock), bees responded to the opportunity cost implied by the probability of choosing the less valuable flower in a similar way that they did to aversive stimuli. The sensorial effort bees made to distinguish between pairs of similar colors when the difference in the reward was larger reflects a trade-off in the information used by honey bees between distance in color cues and differences in reward.

Our results also showed that reversal learning was favored when the more highly rewarded color in the reversal phase was white, specifically when alternative colors were more similar (“near”) (Figure 4B). This result suggests that not only does color distance between pairs of flower colors affect visual discrimination and floral choice, but so does the position of colors in the hexagon (Figure 2B), as it may affect other variables (e.g., salience, contrast), which raises questions about color biases, perceptual asymmetries, or contextual effects that may influence learning flexibility.

The direction of reward change, a gain or loss, was also a key factor in forager honey bees’ decision-making. Here we found that a decrease in rewards elicited a stronger behavioral response from foragers than an increase of the same magnitude did in attachment to the flower color with the greater reward (Figure 3). This behavior indicates that honey bees exhibited loss aversion in decision-making. Negative contrast effect is a well-known behavior shown through the animal kingdom [77], and it has also been reported in honey bees and bumble bees when insects switch flower type, abandon food patches, or refuse to respond (e.g., not extending the proboscis in PER paradigms); the mechanism of this behavior has been explained at the sensory level [78,79] and it has been found to be stimulus-dependent [80], where prior expectations affect future perceptions [81,82]. Our results show that honey bees exhibited loss aversion in decision-making, a behavior described in Prospect theory [83]. The increased number of floral visits exhibited by forager bees during the two first rounds of Treatment III suggest that loss aversion is part of the behavior underlying reverse learning.

Taking together the results presented in this study, we have expanded understanding of the mechanisms underlying ecological food specialization in honey bees. Decision-making for floral choice responded to different factors, including both physical aspects of the floral phenotype (color cues and nectar reward) as well as psychological aspects reflected in the perception of the same absolute values of the difference in reward as gains or losses.

## 5. Conclusions

Our results contribute to the understanding of behavioral flexibility in honey bees, manifested not only at reversal color learning but also at initial color learning. Bees paid more attention to avoid making mistakes in choosing the correct flower color at initial color learning when the difference in reward was larger; the choice occurred between flowers more distinct in color cues, and when one of the floral options decreased in reward value. Given that cognitive attention is physiologically expensive [49], our results suggest a mechanism by which the honey bees might regulate their degree of cognitive investment.

Honey bees used and integrated information perceived from primary floral traits (reward), secondary floral traits (color cues), opportunity cost (economic), and loss aversion (psychological perception) to modulate the degree of accuracy exhibited in color learning.

Honey bees that had high accuracy during initial learning made more mistakes during reversal learning, indicating cognitive costs at reversal learning. Bees responded to physical aspects affecting their ability to discriminate between floral phenotypes and the magnitude of the rewards, and to the psychological prospective perception of the probability of loss in the future.

Extrapolating these results in nature suggests that honey bees have evolved mechanisms to assess different floral traits simultaneously in the processes of decision-making. Therefore, their resulting accuracy in flower specialization may represent not a suboptimal foraging behavior, but an optimal cognitive investment depending on both the options available in the “floral market” and the amount of floral distinctness between co-flowering plants. Complete food specialization may lead to the formation of cognitive representations that make it harder for honey bees to adapt to changing environments. Thus, the partial preferences that were observed in forager honey bees across the different experiments can be explained as cognitive costs in attention invested to make correct choices.

Future studies: The effect of trade-offs between independent variables (i.e., cues and rewards) and on the trade-offs between dependent variables commonly used to characterized behavioral responses in bees (e.g., accuracy and speed) creates an opportunity to simultaneously investigate more factors affecting behavior and the cognitive process underlying the decision-making of forager bees, both in the processes of learning at the initial phase of foraging activity as well as in other scenarios of food exploitation by more experienced bees in which previously gained cognition plays a role.

## Figures and Tables

**Table 1:** The design of Experiments 1 to 8 is shown. Experiments 1 through 8 used flower colors that were separated farther in the bee color space, “far” (distinct). Rewards are shown by treatment and direction of change in reward from Treatment I to Treatment II. Experiments 9 to 16 are a repeat of Experiments 1 to 8, but used flower colors that were less separated in the bee color space, “near” (similar).

Experiment	Change	Treatment I	Treatment II	Treatment III
1	Decrease Δρ% = 10%	30% vs. 30%	30% vs. 20%	20% vs. 30%
2	Decrease Δρ% = 20%	40% vs. 40%	40% vs. 20%	20% vs. 40%
3	Decrease Δρ% = 30%	50% vs. 50%	50% vs. 20%	20% vs. 50%
4	Decrease Δρ% = 40%	60% vs. 60%	60% vs. 20%	30% vs. 20%
5	Increase Δρ% = 10%	20% vs. 20%	30% vs. 30%	20% vs. 30%
6	Increase Δρ% = 20%	20% vs. 20%	40% vs. 20%	20% vs. 40%
7	Increase Δρ% = 30%	20% vs. 20%	50% vs. 20%	20% vs. 50%
8	Increase Δρ% = 40%	20% vs. 20%	60% vs. 20%	20% vs. 60%

**Table 2:** MANOVA analyses. Significant factors and interactions affecting flower choice of individual honey bees (*Apis mellifera*) along the 16 experiments conducted in this study.

Source	F	*p* Value
(color)	F_1308_ = 55.3	* p * < 0.0001
(color distinctness)	F_1308_ = 8.2	* p * = 0.0044
(reward difference)	F_1308_ = 5.1	* p * = 0.0242
(color) × (color distinctness)	F_1308_ = 5.6	* p * = 0.0183
(color) × (method)	F_1308_ = 12.0	* p * = 0.0006
(color) × (method) × (reward difference)	F_1308_ = 5.5	* p * = 0.0192
(treatment) × (color)	F_2307_ = 2515.4	* p * < 0.0001
(treatment) × (color distinctness)	F_2307_ = 16.4	*p* < 0.0001
(treatment) × (method)	F_2307_ = 9.7	* p * < 0.0001
(treatment) × (color) × (method)	F_2307_ = 7.2	* p * = 0.0009
(treatment) × (color) × (reward difference)	F_2307_ = 29.4	* p * < 0.0001
(treatment) × (color distinctness) × (reward difference)	F_2307_ = 3.5	* p * = 0.0302
(treatment) × (color) × (method) × (reward difference)	F_2307_ = 3.3	* p * = 0.0368
(treatment) × (color) × (color distinctness) × (reward diff.)	F_2307_ = 3.59	* p * = 0.0287

## Data Availability

The original contributions presented in this study are included in the article/Supplementary Material. Further inquiries can be directed to the corresponding author.

## Supplementary Materials

The following supporting information can be downloaded at https://www.mdpi.com/article/10.3390/insects16090884/s1.
